# Efficacy and safety of antiparasitic therapy for neurocysticercosis in rural Tanzania: a prospective cohort study

**DOI:** 10.1007/s15010-023-02021-y

**Published:** 2023-03-24

**Authors:** D. Stelzle, C. Makasi, V. Schmidt, C. Trevisan, I. Van Damme, C. Ruether, P. Dorny, P. Magnussen, G. Zulu, K. E. Mwape, E. Bottieau, C. Prazeres da Costa, U. F. Prodjinotho, H. Carabin, E. Jackson, A. Fleury, S. Gabriël, B. J. Ngowi, A. S. Winkler

**Affiliations:** 1 Center for Global Health, TUM School of Medicine, Technical University of Munich (TUM), Munich, Germany; 2 National Institute for Medical Research, Muhimbili Medical Research Centre, Dar es Salaam, Tanzania; 3 Centre for Global Health, Institute of Health and Society, Faculty of Medicine, University of Oslo, Oslo, Norway; 4 Department of Translational Physiology, Infectiology and Public Health, Faculty of Veterinary Medicine, Ghent University, Merelbeke, Belgium; 5 Department of Biomedical Sciences, Institute of Tropical Medicine, Antwerp, Belgium; 6 Service of Foodborne Pathogens, Sciensano, Brussels, Belgium; 7 Department of Neuroradiology, Faculty of Medicine, Technical University of Munich, Munich, Germany; 8 Faculty of Health and Medical Sciences, University of Copenhagen, Copenhagen, Denmark; 9 Ministry of Health, Lusaka, Zambia; 10 Department of Clinical Studies, School of Veterinary Medicine, University of Zambia, Lusaka, Zambia; 11 Department of Clinical Sciences, Institute of Tropical Medicine, Antwerp, Belgium; 12 Institute for Medical Microbiology, Immunology and Hygiene, Center for Global Health, Technical University of Munich, Munich, Germany; 13 Département de Pathologie et Microbiologie, Faculté de Médecine Vétérinaire, Université de Montréal, Saint-Hyacinthe, QC J2S 2M2 Canada; 14 Département de Médecine Sociale et Préventive, École de Santé Publique de l’université de Montréal, Montréal, QC H3N 1X9 Canada; 15 Centre de Recherche en Santé Publique (CReSP) de l’université de Montréal et du CIUSS du Centre Sud de Montréal, Montréal, QC H3N 1X9 Canada; 16 Groupe de Recherche en Épidémiologie des Zoonoses et Santé Publique (GREZOSP), Université de Montréal, Saint-Hyacinthe, QC J2S 2M2 Canada; 17 Instituto de Investigaciones Biomédicas-UNAM/Instituto Nacional de Neurología y Neurocirugía/Facultad de Medicina-UNAM, Ciudad de Mexico, Mexico; 18 University of Dar es Salaam, Mbeya College of Health and Allies Sciences, Mbeya, Tanzania; 19 German Center for Infection and Research (DZIF), Munich, Germany; 20 Department of Neurology, TUM School of Medicine, Technical University of Munich (TUM), Munich, Germany

**Keywords:** *Taenia solium*, Neurocysticercosis, Neglected tropical diseases, Antiparasitic medication, Albendazole, Praziquantel

## Abstract

**Purpose:**

Neurocysticercosis is common in regions endemic for *Taenia solium*. Active-stage neurocysticercosis can be treated with antiparasitic medication, but so far no study on efficacy and safety has been conducted in Africa.

**Methods:**

We conducted a prospective cohort study on treatment of neurocysticercosis in Tanzania between August 2018 and January 2022. Patients were initially treated with albendazole (15 mg/kg/d) for 10 days and followed up for 6 months. Additionally in July 2021, all participants who then still had cysts were offered a combination therapy consisting of albendazole (15 mg/kg/d) and praziquantel (50 mg/kg/d). Antiparasitic treatment was accompanied by corticosteroid medication and anti-seizure medication if the patient had experienced epileptic seizures before treatment.

**Results:**

Sixty-three patients were recruited for this study, of whom 17 had a complete follow-up after albendazole monotherapy. These patients had a total of 138 cysts at baseline, of which 58 (42%) had disappeared or calcified by the end of follow-up. The median cyst reduction was 40% (interquartile range 11–63%). Frequency of epileptic seizures reduced considerably (*p < *0.001). Three patients had all active cysts resolved or calcified and of the remaining 14, eight received the combination therapy which resolved 63 of 66 cysts (95%). Adverse events were infrequent and mild to moderate during both treatment cycles.

**Conclusion:**

Cyst resolution was unsatisfactory with albendazole monotherapy but was very high when it was followed by a combination of albendazole and praziquantel.

**Supplementary Information:**

The online version contains supplementary material available at 10.1007/s15010-023-02021-y.

## Introduction

Neurocysticercosis (NCC), caused by larvae of the zoonotic tapeworm *Taenia solium*, is common in people with epilepsy in low-income and middle-income countries where sanitation is poor and pigs roam in search for food [1–3]. Studies have shown that in areas highly endemic for *T. solium*, around 30% of people with epilepsy suffer from NCC, with wide variation even between communities in the same area [4–9]. Epileptic seizures are the most common, but not the only neurological sign/symptom. Severe headache or focal neurological deficits are other common neurological signs/symptoms of NCC [10, 11].

NCC lesions go through a cycle of four different stages vesicular, colloidal, granular-nodular and nodular-calcified stage [12]. The first three are active stages (viable stage: vesicular lesions; degenerating stage: colloidal and granular-nodular lesions), while the nodular-calcified stage is considered as inactive. Patients are mostly symptomatic in the degenerating (colloidal and the granular-nodular) stage but can also be or become symptomatic in the other stages [13–15].

NCC lesions may naturally evolve through the above stages and eventually calcify or disappear without calcification; a process that can take several years which can be sped up by antiparasitic medication. In general, treatment recommendations for NCC depend on the stage, number and the location of lesions, i.e., whether they are in the brain parenchyma or in the extraparenchymal space. Currently, two sets of NCC management guidelines are in place—one by the World Health Organization (WHO) and one by the Infectious Diseases Society of America (IDSA) together with the American Society of Tropical Medicine and Hygiene (ASTMH). Although both guidelines are similar, they differ in some aspects [16, 17]. For extraparenchymal NCC, the IDSA/ASTMH guidelines recommend removal of the cysts by minimally invasive neurosurgical endoscopy if lesions are intraventricular, and antiparasitic treatment if cysts are located in the subarachnoid space (basal cisterns or Sylvian fissure) [17]. The WHO guidelines do not make any recommendations for treatment of extraparenchymal NCC [16]. For parenchymal NCC, the WHO guidelines recommend albendazole or praziquantel monotherapy depending on availability of drugs [16]. IDSA/ASTMH recommend two different antiparasitic treatment regimens depending on the number of lesions. For patients with one or two active lesions, albendazole (15 mg/kg/d) monotherapy is recommended for 10 to 14 days, accompanied by corticosteroid medication and, if necessary, anti-seizure medication (ASM). For patients with more than two lesions, a combination therapy of albendazole (15 mg/kg/d) and praziquantel (50 mg/kg/d) is recommended, in addition to corticosteroids and ASM. These recommendations are primarily based on results from two clinical trials by the Cysticercosis Working Group in Peru, and expert opinions [18, 19]. In both trials, significantly better efficacy of combination therapy compared with albendazole monotherapy in terms of cyst resolution was found only in patients with more than two active-stage lesions. Whilst there was only one further study assessing combination therapy for paediatric NCC [20], experts confirmed the superiority of combination therapy from their treatment of patients outside research settings [21–26]. For albendazole monotherapy, a recent meta-analysis reported resolution of all cysts in 41% (95%CI 36–47%) of patients and a cyst degeneration of 81% (95%CI 70–94%) [27].

So far, no study on antiparasitic therapy in patients with NCC has been conducted in sub-Saharan Africa. Therefore, we aimed to conduct the first sub-Saharan African study on treatment efficacy and safety of albendazole monotherapy and albendazole and praziquantel combination therapy in patients with active, symptomatic NCC; cyst resolution at imaging represented the primary outcome and reduction of symptom frequency the secondary outcome. We also assessed the feasibility of anti-parasitic treatment in patients with NCC in a rural African setting.

## Methods

### Patient recruitment and eligibility criteria

We conducted a prospective cohort study of patients with active NCC in rural southern Tanzania consisting of two parts: the albendazole monotherapy treatment study within the SOLID project and the albendazole and praziquantel combination therapy treatment study TOPANA. For the albendazole monotherapy treatment study patients were recruited through the SOLID project from three district hospitals (Ifisi, Tukuyu and Vwawa) between August 2018 and April 2020. These district hospitals were chosen because they are in areas endemic for *T. solium* and because the infrastructure around Mbeya city, in particular the laboratory facilities, were well suited for the study requirements. The SOLID project aimed at assessing a novel point-of-care test for the diagnosis of *T. solium* taeniasis and (neuro)cysticercosis. The protocol of the SOLID project has been published elsewhere [28]. NCC diagnosis was made following the latest Del Brutto criteria, by a combination of neuroimaging (computed tomography [CT]) and laboratory testing (Antigen ELISA, LLGP-EITB, and rT24H-EITB) [29]. CT scans were interpreted by two independent readers (CR, neuroradiologist; AF, clinical expert for NCC). In case of disagreement, a third reviewer (ASW, consultant neurologist) adjudicated the case. The number of cysts (with and without scolex), stage (vesicular, colloidal-vesicular, granular-nodular and calcified) and location of lesions (intraparenchymal or extraparenchymal) were assessed. All patients with at least one NCC lesion in the vesicular stage were eligible for the treatment study within the SOLID project. Symptomatic patients and patients without contraindications for antiparasitic treatment, were eligible for treatment with albendazole monotherapy. All other patients (i.e., asymptomatic patients, patients with contraindications for treatment and patients who refused treatment) underwent follow-up within clinical routine care without antiparasitic treatment.

“Symptomatic” was defined as ever having had epileptic seizures or severe progressive headache. Severe progressive headache was defined as a severe headache that interferes with the patient’s daily activities and that gets progressively worse over time. The definition of epilepsy and severe progressive headache was made by the neurologists/general medical doctors within the team (DS, CM, AF, GZ, BJN, ASW) according to current research standards. Asymptomatic patients (i.e., patients not eligible for treatment), patients with contraindications for treatment or patients who refused treatment were also included and were followed-up without treatment to assess the natural course of disease.

### Treatment: albendazole monotherapy

The initial antiparasitic treatment was albendazole 15 mg/kg/d (up to 1200 mg/d in two doses) for 10 days accompanied by oral corticosteroid therapy (dexamethasone 12 mg/d in one dose if less than 10 cysts, otherwise 20 mg/day in one dose; the higher dose was chosen for patient safety reasons) to suppress inflammation caused by degenerating cysts in the brain and the resulting inflammatory response. Corticosteroid therapy started one day prior to albendazole therapy with a single loading dose of 16 mg. After the end of albendazole treatment, dexamethasone therapy was tapered for 3 days with half the dosage per day. Also, all patients with epileptic seizures were continued on ASM. If necessary, ASM was optimized before therapy to control epileptic seizures. Patients who still had active NCC lesions after the re-evaluation in July 2021 were treated with a combination of albendazole (15 mg/kg/d) and praziquantel (50 mg/kg/d) for 10 days. Based on the experience from the first treatment round (albendazole monotherapy) with only few adverse events and good tolerability of medication, 12 mg dexamethasone was given to every patient irrespective of the number of active NCC lesions. Alike albendazole monotherapy, all patients with seizures were continued on ASM which was optimized before therapy, if necessary.

Before treatment initiation, patients were tested for HIV (blood), tuberculosis (sputum), *Strongyloides* spp. infections (faeces) and underwent fundoscopy for the exclusion of ocular NCC according to the recommendations by IDSA/ASTMH [17]. Furthermore, patients were examined (general medical examination and neurological examination) to exclude contraindications for treatment. Medication was handed out to the patient every day by trained nursing staff and intake was directly observed. First, only albendazole and dexamethasone were handed out by the nursing staff, and patients were told to take their ASM as usual. However, since one of the first treated patients had two epileptic seizures during hospitalisation because of not having taken their ASM, ASM was subsequently also prepared and handed out by the nursing staff. Patients were hospitalised for the entire treatment cycle, but if patients wanted to leave after day 7 of the treatment cycle, they had to sign a document stating that they left against medical advice; these patients had to come to the hospital every day to pick up their medication and intake thereof was observed. Blood glucose level and blood pressure were measured every day and any adverse events of treatment were recorded.

### Follow-up of patients receiving albendazole monotherapy

Patients who were treated with antiparasitic medication were followed up 6 weeks and 6 months after treatment termination. Due to the COVID-19 pandemic, follow-up was interrupted in March 2020. Hence, in July 2021, all patients treated with albendazole monotherapy were invited for an additional follow-up visit including CT scanning. This follow-up period lasted on average 28 months after the completion of the initial treatment (range 16–35 months). At each follow-up visit, adverse events of therapy, occurrence of epileptic seizures or severe headache episodes were recorded, patients were examined, and CT scanning (± contrast) was conducted.

### Recruitment, eligibility, follow-up and treatment of patients with combination therapy

Patients who still had vesicular NCC lesions at the July 2021 evaluation were offered a second treatment cycle within an observational study called TOPANA, in which the efficacy of a combination of albendazole and praziquantel was evaluated. The combination therapy consisted of albendazole (15 mg/kg/d) and praziquantel (50 mg/kg/d). Patients receiving the combination therapy were followed up 6 weeks and 6 months after treatment termination and had the same evaluations as in the SOLID project. Patients receiving combination therapy were also asked to answer two quality of life questionnaires (see details below).

### Outcomes

The primary outcome of this study was the resolution of cysts which was defined as complete disappearance of cysts or as calcification of lesions at long-term follow-up. Long-term follow-up was defined as either 6-month follow-up, or the follow-up conducted in July 2021. This combination was chosen because some patients had their regular 6-month follow-up before the study interruption and others only had the follow-up in July 2021. For patients (*n = *7) present at both timepoints, the July 2021 follow-up was used. Three patients were only present at the July 2021 follow-up but did not have a regular 6-month follow-up; seven patients only had a regular 6-month follow-up and were not present at the July 2021 follow-up. The secondary outcomes were reduction of symptoms associated with NCC, defined as reduction of the number of epileptic seizures per year and reduction of the number of episodes of severe progressive headache. For the number of epileptic seizures per year, the frequency was extrapolated by multiplying the frequency per week with 52, the frequency per month with 12 or the number of seizures during 6 months of follow-up by 2.

For the treatment round with combination therapy, the secondary outcome was the change in quality of life between baseline and at the 6-month follow-up. Two questionnaires were used: the QOLIE-31 and the WHOQOL-BREF questionnaire. Both questionnaires are well validated and have been published elsewhere [30, 31]. They were first translated into Kiswahili and then back-translated to English by native Kiswahili speakers. The QOLIE-31 is a short version of the QOLIE-89 and assesses epilepsy-specific quality of life with 31 questions; one question is a general question on quality of life and the remaining 30 questions are summarised into the following seven sub-categories (each ranges from 0 to 100) which are combined to an overall score that ranges from 0 to 100: seizure worry (5 questions), overall quality of life (2 questions), emotional well-being (5 questions), energy/fatigue (4 questions), cognitive function (6 questions), medication effect (3 questions) and social function (5 questions). The WHOQOL-BREF is a short version of the WHOQOL-100 and consists of 26 questions which are grouped into four categories of quality of life: physical health, psychological health, social relationships, and environment. The categories are not epilepsy specific. The scores of each category range from 4 to 20.

### Statistical analyses

The study followed a convenience sampling of all consecutive patients with active, symptomatic NCC recruited through the SOLID project. The sample size was determined for the main objective of the SOLID project and the assumptions can be found in the published protocol [28]. Descriptive statistics of number, location and stage of lesions were reported at baseline, and at long-term follow-up for patients treated and not treated with antiparasitic medication. Furthermore, these statistics were also reported at 6-week and 6-month follow-up for the subset of patients who were present at all follow-up timepoints. The medians and interquartile ranges of the change in the number of lesions by location and the change in frequency of epileptic seizures were reported (from baseline to 6-week, 6-month, and long-term follow-up). Furthermore, these changes were compared from baseline to long-term follow-up using paired Wilcoxon tests. Changes in quality-of-life scores from baseline to 6 months after treatment with combination therapy were assessed using paired Wilcoxon test. We report descriptive statistics but refrained from statistical testing of comparisons between treated and not treated patients because patients were not randomised to treatment but were selected based on symptoms and (contra-)indications for antiparasitic treatment. We did not run statistical tests either for differences in the efficacy of albendazole monotherapy and combination therapy because all patients treated with combination therapy had received albendazole monotherapy before (even though it was at least 16 months [mean 28 months] before). The change from baseline to 6 weeks and to 6 months after treatment was analysed for the subset of people with all follow-up timepoints available. Only patients with long-term follow-up were included in the analysis. A type I error of 5% was used as a threshold for statistical significance.

### Ethical considerations

All patients were informed about all parts of the two treatment studies, the treatment study within the SOLID project and the TOPANA treatment study (see Methods), and signed an informed consent before inclusion. The patients who were included in both the SOLID project and the TOPANA study signed a separate informed consent of each of the two treatment studies. Both treatment studies received ethical approval by the Klinikum rechts der Isar (Technical University of Munich, Germany) Ethical Committee (299/18S for albendazole monotherapy within the SOLID project; 33/19S for combination therapy within the TOPANA study), by the National Ethics Health Research Committee (NatREC) of Tanzania (NIMR/HQ/R.8a/Vol.IX/2597 for SOLID and NIMR/HQ/R.8c/Vol.I/1808 for TOPANA), by the Ethics Committee of the Institute of Tropical Medicine, Antwerp, Belgium (IRB/AB/ac/112 Ref 1177/17) and that of the University of Antwerp, Belgium (EC UZA 17/31/352), both for the SOLID project. The SOLID project including the treatment of symptomatic NCC patients with albendazole monotherapy was registered in the Pan African Trials Registry (PACTR201712002788898). The TOPANA study for the treatment of symptomatic NCC patients with albendazole and praziquantel combination therapy was registered on ClinicalTrials.gov (Identifier: NCT03834337). The reporting of this current study, consisting of the two treatment studies as described above (see Methods), followed the STROBE Statement (see Supplement).

## Results

### Target and study population

**Fig. 1:**
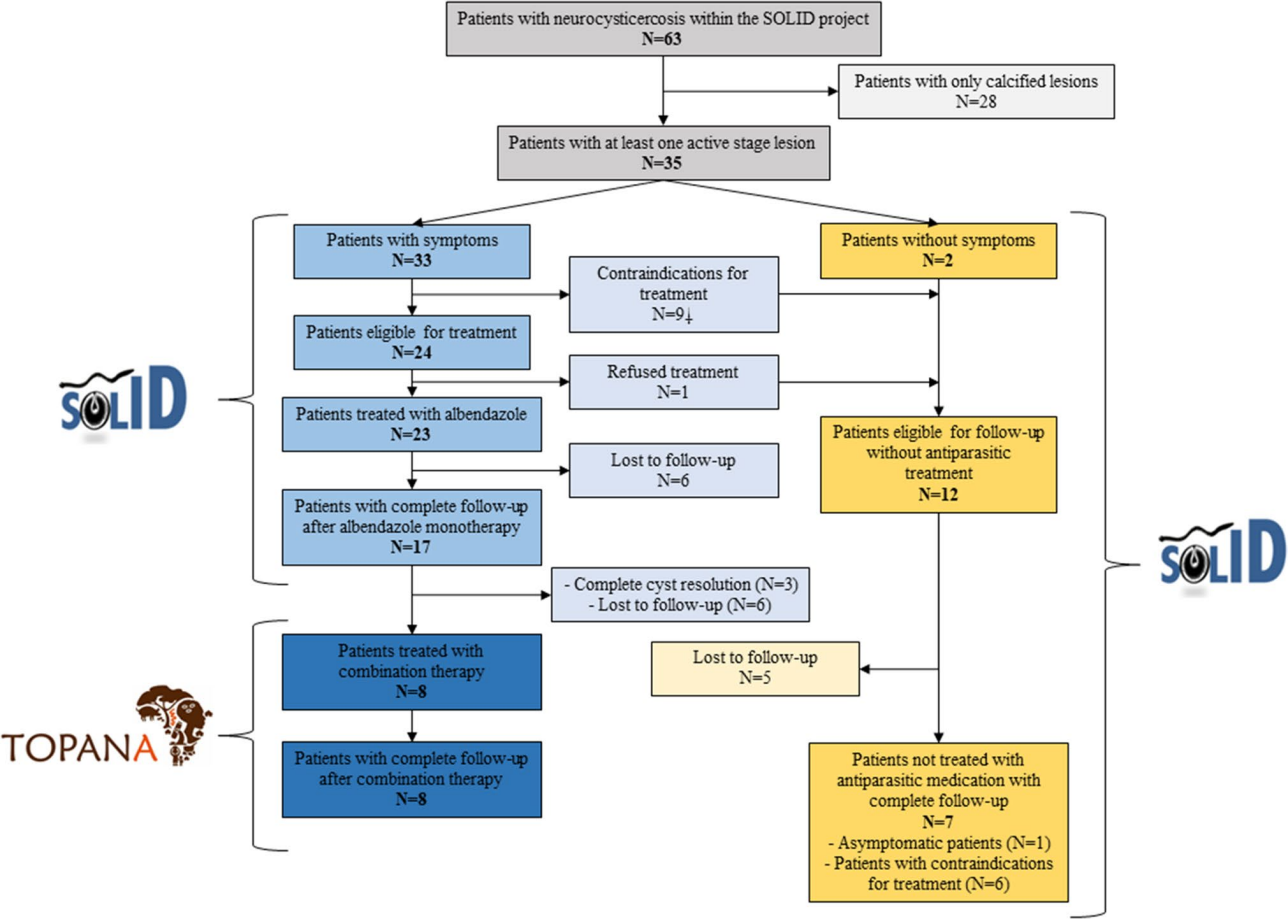
Flowchart of the study.  Eight patients with large number of lesions, one patient with lesion in the fourth ventricle

Overall, 63 patients were diagnosed with NCC between August 2018 and April 2020 in the SOLID project (Fig. 1). Thirty-five of these people had at least one vesicular stage lesion, of whom 33 were symptomatic. Nine patients were not eligible for antiparasitic treatment because of the large number of lesions (range 28–83, *n = *8) or because lesions were in critical areas (i.e., in the fourth ventricle, *n = *1), putting them at high risk of severe adverse events, leaving 24 eligible for treatment. Twenty-three patients received antiparasitic treatment since one eligible patient refused it. Asymptomatic patients (*n = *2), patients with contraindications for treatment (*n = *9) or patients who refused treatment (*n = *1) were eligible for follow-up without antiparasitic treatment (Fig. 1). Seventeen of the 23 treated patients and seven of 12 patients not treated with antiparasitic medication had a long-term follow-up and were included in the analyses. The others were excluded from the analysis. The characteristics of included patients are summarized in Table 1.

**Table 1:** Baseline characteristics of included patients with active symptomatic NCC who were treated with albendazole monotherapy or not treated with antiparasitic medication between August 2018 and April 2020, Tanzania

	Treatment group-albendazole monotherapy (%)	Non-treatment group (%)
Number	17	7
Sex		
Female	6 (35)	3 (43)
Male	11 (65)	4 (57)
Age		
Median [IQR]	42 [32–55]	50 [37.5–53.5]
Range	27–68	26–60
Occupation		
Smallholder farmer	16 (94)	5 (71)
Other	1 (6)	2 (29)
Recruitment		
Ifisi	1 (6)	1 (14)
Tukuyu	6 (35)	0 (0)
Vwawa	10 (59)	6 (86)
Headache	6 (35)	3 (43)
Seizures	16 (94)	6 (86)
Age of onset of seizures		
Median [IQR]	28 [21.3–38.3]	43.5 [40–47]
Time since onset of seizures		
Median [IQR]	13 [7.3–23]	7 [3–8.8]
Anti-seizure medication	16/16 (100)	2/6 (33)
Onset of seizures		
Focal	15 (94)	4 (67)
Generalised	1 (6)	2 (33)
Anti-seizure medication type		
Phenobarbitone	4 (25)	0
Carbamazepine	1 (6)	0
Phenytoin	1 (6)	0
Phenobarbitone + Carbamazepine	9 (56)	1 (50)
Phenobarbitone + Phenytoin	1 (6)	1 (50)
Seizure frequency		
Daily to weekly	0	0
Weekly to monthly	5 (31)	0
Monthly to yearly	6 (38)	5 (83)
Less than yearly	5 (31)	1 (17)
Median per year	4 [0.5–12]	2.5 [0.8–4]

*IQR* interquartile range

### Characteristics of the seventeen included patients who received antiparasitic treatment

The median age of treated patients was 42 years (interquartile range [IQR] 32–55 years); eleven (65%) were male, and all but one (94%) were smallholder farmers. On average, patients had 8.1 active-stage lesions (median 5; IQR 1 to 11); five patients had only one single active-stage lesion. Overall, these patients had 138 active lesions, of which 39 (29%) were located in the extraparenchymal space (all in the subarachnoid space and sulci around the cortex; none in the ventricles or basal cisterns), and 99 (71%) in the brain parenchyma. Ninety-six lesions contained a scolex and a total of 258 (median 14; IQR 8 to 20) calcifications were seen on the CT scans (Tables 1 and 2).

**Table 2:** Cyst resolution at baseline and long-term follow-up in patients receiving albendazole monotherapy

	Baseline *N = *17			Long-term follow-up *N = *17			Lesion reduction	
	Sum	Median [IQR]	Mean	Sum	Median [IQR]	Mean	Median (IQR)	*p* value (Wilcox)
Total active lesions	138	5 [1–11]	8.1	80	3 [1–6]	4.7	40% (11–63%)	0.14
Active parenchymal lesions	99	3 [1–6]	5.8	58	1 [1–3]	3.4	25% (0–45%)	0.36
Active extraparenchymal lesions	39	1 [0–4]	2.3	22	0 [0–2]	1.3	50% (24–100%)	0.28
Number of cysts with scolex	96	4 [1–5]	5.6	62	1 [0–5]	3.6	45% (13–100%)	0.23
Calcifications	258	14 [8–20]	15.2	260	17 [8–20]	15.3	0% (0–0%)	0.92
NCC with active lesions		17			14			
Epileptic seizures	16			4^b^				
Median [IQR] per year^a^		4 [0.5–12]			0 [0–1]		100% (50–100%)	< 0.001

^a^Extrapolated

^b^Number of patients who had epileptic seizures during follow-up

Sixteen of the 17 treated patients had been suffering from epileptic seizures for a median of 13 years. All patients had epileptic seizures with generalisation, 15 (94%) with unilateral onset and with a median number of four seizures per year; all were on ASM. Six patients reported frequent headache episodes, most commonly with a strength of 3 on a scale from 0 (no pain) to 5 (worst pain imaginable).

### Characteristics of eligible patients who did not receive antiparasitic treatment

Seven patients were not treated with antiparasitic medication and were only followed up. These patients were in the median 50 years old (IQR 37.5–53.5 years), four were male (57%) and five were smallholder farmers (71%). These patients had in total 183 and on average 26.4 active-stage lesions (median 20; IQR 9–47), 19 in the parenchyma (median 11; IQR 7–31.5) and 7.4 in the extraparenchymal space (cortical sulci, median 11, IQR 2–11.5). Six of the not treated patients had epileptic seizures (in the median for 7 years) and two were on ASM (Table 1 and STable 1).

### Follow-up of patients treated and not treated with albendazole monotherapy

**Fig. 2:**
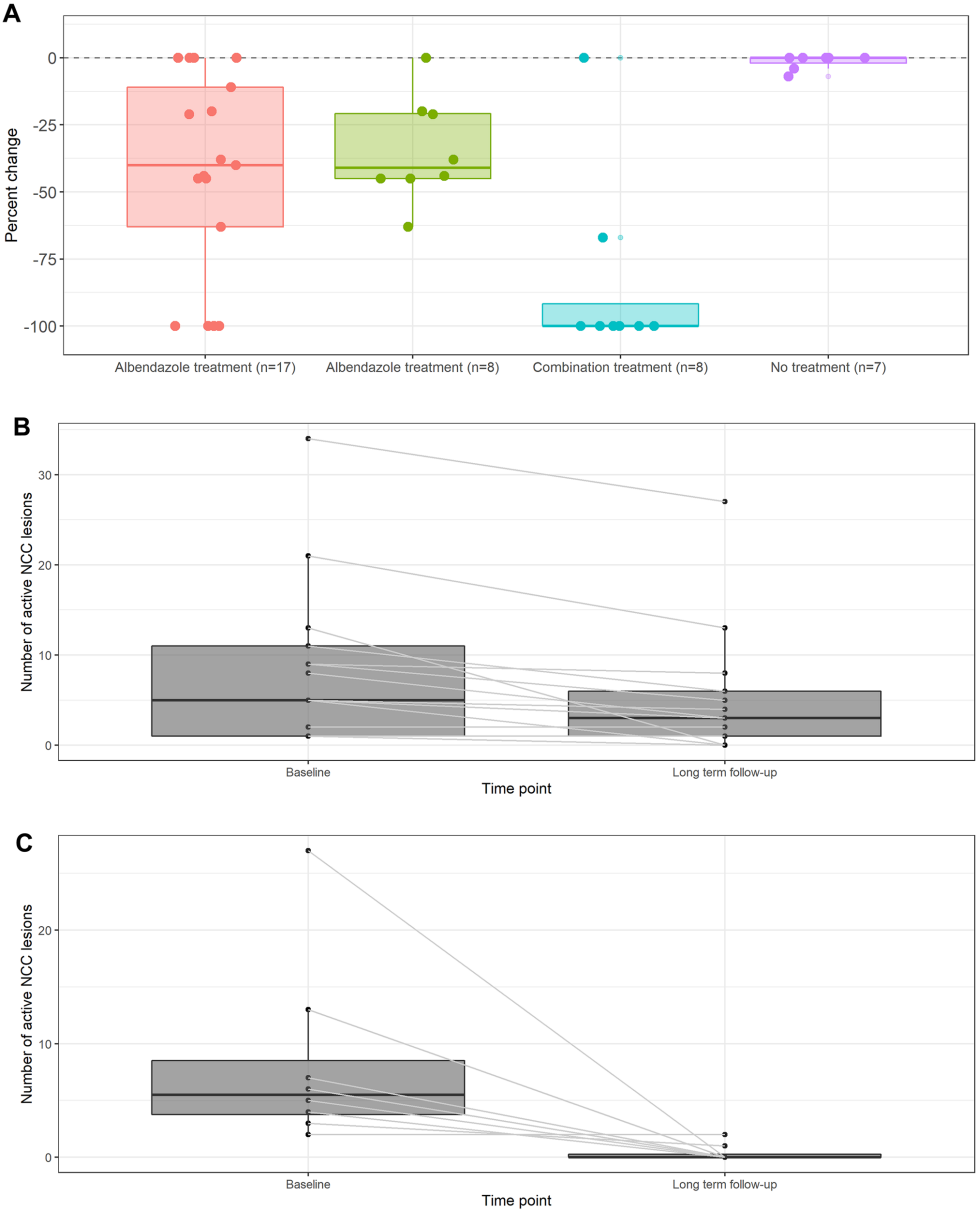
**A** Treatment efficacy in terms of cyst resolution by treatment regimen, and natural disease progression without antiparasitic treatment^a. a^Albendazole treatment (*n = *8) refers to the eight patients who received first a treatment cycle with albendazole and because of incomplete cyst resolution afterwards treatment cycle with combination therapy. They are a subset of the 17 patients on the left. **B** Cyst resolution in the seventeen patients treated with albendazole monotherapy. **C** Cyst resolution in the eight patients treated with combination therapy

At long-term follow-up, 58/138 (42%) and 3/183 (2%) of cysts had resolved in patients treated with albendazole monotherapy and not treated, respectively. The median reduction in the number of active cysts per patient was 40% (interquartile range 11% to 63%) in patients who were treated and 0% (interquartile range 0–4%, Table 2 and Fig. 2A) in patients who were not treated (S Table 1). At long-term follow-up all lesions had resolved in two of five (40%) treated patients with single lesions, and one of twelve (8%) patients with more than one vesicular lesion. Medians of 25% and 50% of parenchymal and extraparenchymal lesions resolved during the long-term follow-up period, respectively. The eleven patients who were present at the 6-week and 6-month follow-up had a cyst resolution of 28/78 (36%) cysts after 6 weeks, and 32/78 (41%) after 6 months (Fig. 2B, S Table 2, and S Fig. 1). Seven patients were present at a 6-month follow-up and the follow-up in July 2021; four of them did not have a change in the number of active lesions, and three patients had one cyst resolved in total. Overall, the median frequency of epileptic seizures reduced from 4 [IQR 0.5–12] before to 0 [IQR 0–1] per year (*p < *0.001) after albendazole monotherapy and only four patients experienced epileptic seizures during follow-up.

### Follow-up of patients treated with combination therapy

**Fig. 3:**
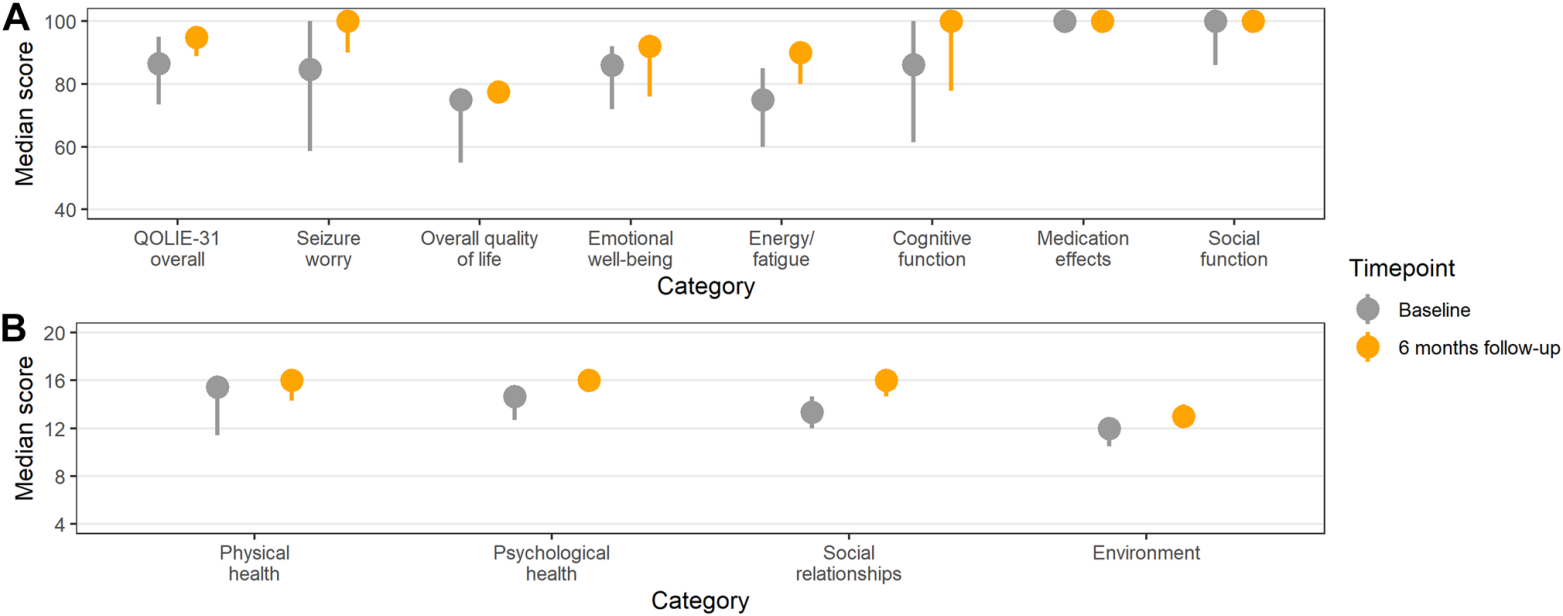
Change in quality of life from before and 6 months after combination therapy (**A** QOLIE-31, **B** WHOQOL-BREF)^a. a^Median (point) and range of values (lines)

Eight patients received treatment with combination therapy in July 2021 after re-evaluation. These patients showed a median cyst resolution of 41% (interquartile range 20–45%; overall resolution of 35/101 active lesions) at long-term follow-up after receiving albendazole monotherapy. After treatment with the combination therapy at 6-week follow-up, 61/66 lesions (92%) had resolved and at 6-month follow-up, 63/66 lesions (95%) had resolved. Six out of eight patients had no active lesions anymore (Fig. 2A–C, S Fig. 1 and S Table 3). The overall epilepsy-specific quality of life measured by the QOLIE-31 increased from a median score of 87 (IQR 80–89) to a score of 95 (IQR 93–95; *p = *0.02; Fig. 3 and S Table 4). An increase in quality of life was observed in all sub-categories of the QOLIE-31 (apart from medication effects and social function which were 100 already at baseline). The category with the lowest baseline score was energy/fatigue (75 points, IQR 71–80); for this category, and also for seizure worry and cognitive function large increases of more than ten points were observed. An increase was also observed in all four WHOQOL-BREF categories, but only psychological health, social relationships and environment were statistically significant.

### Safety and adverse events with albendazole monotherapy and combination therapy

With albendazole monotherapy, one patient with epilepsy had two epileptic seizures within 2 h on day 5 of the treatment cycle. None of the patients had headache during therapy and no other local or systemic adverse events were observed. Blood pressure and fasting blood glucose levels remained stable within normal range throughout the treatment cycle. Of the eight patients who received treatment with a combination of albendazole and praziquantel, three reported a headache episode, which in one patient lasted for 3 days. Alike albendazole monotherapy, blood pressure and fasting blood glucose levels remained stable and mainly within the normal range throughout the treatment cycle (S Figs. 2 and 3).

## Discussion

This is the first study on systematic antiparasitic treatment in people with active symptomatic NCC in sub-Saharan Africa. In Tanzanian patients, we found cyst resolution with albendazole monotherapy to be slightly lower than previously reported in studies of Latin American and South-East Asian populations [27]. However, in most patients who received albendazole monotherapy, not all vesicular cysts resolved. Subsequent combination therapy of albendazole and praziquantel, on the other hand, rendered nearly all patients vesicular cyst-free. Of note, most cysts had been resolved within 6 weeks after the combination therapy. Patients who received either single or combination therapy also reported fewer epileptic seizures after therapy. Quality of life, especially in terms of energy and fatigue, improved after combination therapy.

In our study, treatment was safe and there were hardly any adverse events apart from mild to moderate headache, which resolved with anti-inflammatory medication. In the trial in Peru, severe adverse events were also reported to be rare [18]. However, it is important to point out that our study was conducted under strict research conditions and treatment was supervised by a study team on site (with a neurology consultant stand-by). This situation is not comparable to the current treatment situation in rural sub-Saharan Africa, where there are very few trained specialists. This needs to be considered when recommending treatment regimens for this region. It should be noted that although adverse events seem to be rare, they can be severe and potentially lethal. Hence, close supervision is crucial to detect clinical deterioration as early as possible. We, therefore, recommend that patients should be hospitalised throughout the entire treatment cycle, a requirement that is hard to meet under local circumstances, as people are usually only admitted to hospital for severe illness. Also, family members usually accompany patients and take care of them during their admission. Therefore, hospitalisation has a major financial and social impact on the patient’s family, and it is unlikely that patients can be motivated to stay in a ward only for safety of treatment.

In our study, treatment was generally well tolerated. Only one patient had epileptic seizures during therapy, because he had not taken his ASM. As this patient was among the first four patients treated, after the incident, ASM was given to all patients and intake was also observed. There were also no metabolic side effects of the treatment, as expected. With combination therapy, mild to moderate headache was a fairly common adverse event. Although this is a known common side effect of praziquantel, we paid particular attention to it as it may reflect increased intracranial pressure due to focal or generalised cerebral oedema or hydrocephalus in the light of a potentially more severe inflammatory process during combination therapy. Magnetic resonance imaging (MRI) was also not available, so there was the risk of undetected intraventricular cysts that could block cerebrospinal fluid circulation and be life-threatening. We, therefore, observed these patients closely and performed fundoscopy to detect signs of increased cranial pressure. When treating patients for active symptomatic NCC in sub-Saharan Africa, a number of factors need to be taken into account, especially if only CT and no MRI is available. First, there are hardly any intensive care units and there is only limited access to relevant monitoring devices; second, staff is often not trained in the management of neurological emergencies; and third, treatment options are limited. Overall, clinical monitoring of patients in rural areas of LMIC is challenging.

Another issue to consider when treating people with active symptomatic NCC in sub-Saharan Africa is co-morbidity with HIV/AIDS. In our study, none of the patients was living with HIV, but treatment effect and side effects may be different in people living with HIV. Especially in sub-Saharan Africa, an HIV test should be performed before treatment, as the efficacy of antiparasitic medication may be altered due to a compromised immune response. Similarly, corticosteroid medication may also impact antiparasitic efficacy, blood pressure and blood glucose levels, which are less stable in people living with HIV [32, 33].

To date, only two studies have assessed the effect of combination therapy on active symptomatic NCC in adults [18, 19]. Both trials were conducted in Peru, but experts from other parts of the world have confirmed the superior effect of combination therapy in their daily routine [20–26]. IDSA/ASTMH recommend combination therapy for patients with more than two active-stage lesions. This cut-off was derived from the two Peruvian trials in which patients with one or two active-stage lesions showed a cyst reduction of 63% in both trials (statistically not superior to albendazole monotherapy), but in patients with more than two lesions, cyst reduction was 94% and 99% (and statistically superior to albendazole monotherapy in both studies) [18, 19]. The sample size of our study was too small to assess differences in efficacy between patients with up to and more than two active lesions.

The higher cyst resolution in people treated with combination therapy compared with albendazole monotherapy needs to be interpreted with caution, as only patients who did not have a full cyst resolution with albendazole monotherapy and no treatment naïve patients were treated with combination therapy. It is well known that previous exposure to a particular antigen allows for a better immune response upon a second exposure to the same antigen. The beneficial role of immune memory, which underlies the success of vaccines, has also been shown for other helminth infections such as schistosomiasis [34–36]. It is, therefore, possible that the high efficacy observed in the second treatment cycle may be due to antigen exposure during the first treatment cycle with albendazole, although treatment with combination therapy occurred in the median more than 2 years after albendazole therapy. However, it could also be argued that natural antigen exposure may already have occurred before the first treatment cycle, as all patients had at least four calcified NCC lesions (median 17.5). In this study, we were neither able to evaluate the effect of pre-treatment with albendazole on the efficacy of antiparasitic combination therapy nor the effect of natural disease progression. Previous studies, however, highlighted an increased efficacy of combination therapy over albendazole monotherapy as first-line therapy [18, 19].

In our study, most people reported a considerable reduction of epileptic seizures after albendazole therapy. This could be due to several reasons. First, we adjusted the patients’ ASM regimen before treatment—where necessary—and ensured that patients took their medication for at least the following 6 months. Stock-outs and discontinuation of medication are common in people with epilepsy in sub-Sahara Africa [37–39]. Second, patients with fewer lesions were generally less symptomatic, and although only few patients had all active-stage lesions resolved after albendazole monotherapy, a marked decrease of lesions was observed. Indeed, a previous study described a reduction in seizure frequency after albendazole therapy [40]. However, it would be unlikely that such a large reduction in seizure frequency would be seen in people who did not have a full cyst resolution. In the Peruvian trial, an 81% decrease in seizure frequency was reported for patients with full cyst resolution compared to those who still remained with cysts [18]. Third, the anti-inflammatory effect of antiparasitic and corticosteroid medication could have impacted the pathogenesis of epileptic seizures independently of cyst resolution. The importance of inflammatory processes on epileptogenesis has been demonstrated previously [41, 42]. Last, recall bias may also have played a role. This could have been addressed through the use of seizure diaries, rather than just asking about seizure frequency at follow-up visits. While frequency after therapy could have been assessed using seizure diaries, the frequency before treatment will always be affected by recall bias, which can go either way—an overestimation or an underestimation of the frequency, although overestimation seems more plausible. It is important to note that in the above-mentioned study from Peru, a considerable reduction of epileptic seizures, particularly of those with generalisation, was observed and associated with treatment success [18]. We, therefore, assume that patients truly had fewer seizures after therapy. Also, we observed an improvement in quality of life in people treated with combination therapy. In particular, patients reported feeling more energetic and less fatigued. This finding could have been expected after withdrawal from ASM, but all patients remained on ASM until the end of the study. In addition, a recent study demonstrated that patients with NCC had lower quality of life [43].

### Limitations

Our study had several limitations that may have partially influenced our results. Firstly, we merged cyst resolution of patients with 6-month follow-up and those with a longer follow-up. This may have led to an overestimation of the true treatment effect of albendazole, but we do not believe that this significantly influenced our results, as most of the treatment effect was generally observed within six weeks after therapy, and the natural disease progression is generally very slow, as we observed in our small group of non-treated patients. Other studies also used 6 months as their endpoint, because no treatment effect of antiparasitic therapy is expected to occur thereafter. In the patients with a follow-up at 6 months and in July 2021 (long-term), no cyst reduction beyond the natural disease progression was observed. Another limitation of our study was associated with the comparison of people who received antiparasitic treatment with those who did not. In this way, we wanted to study both the treatment effect and the natural disease progression. However, we neither randomised them into groups, nor were they similar in their disease presentation, as only those patients who refused therapy or had too many cysts or cysts in critical locations were not eligible for antiparasitic therapy. Loss to follow-up was also a problem, especially in the group of patients not treated with antiparasitic medication. This is a common problem in resource-limited settings where participation in CT examination can require substantial travel; for some patients in our study from remote areas, this was almost a day's journey. Nevertheless, we consider this comparison important, as otherwise we would not have been able to establish the natural disease progression. However, we established the natural disease progression only in a specific group of patients with many lesions or contraindications for antiparasitic treatment. Another limitation concerns the treatment effect of combination therapy. Based on our study design, we offered the combination therapy to patients who had previously been treated with albendazole alone and for whom this treatment failed. While it is likely that treatment with combination therapy would also have been successful in patients who were successfully treated with albendazole alone, we cannot determine this. Therefore, we cannot say with certainty that combination therapy is more efficacious than albendazole therapy alone, as we did not treat anybody with two cycles of albendazole monotherapy or with combination therapy as first-line therapy.

## Conclusions

Treatment with a combination of albendazole and praziquantel in people with active symptomatic NCC after treatment with albendazole monotherapy showed excellent results. In contrast, albendazole monotherapy performed less well than expected. Treatment was safe under research conditions and under directly observed therapy, which differs considerably from everyday treatment conditions in sub-Saharan Africa. Treatment logistics, which can have a direct impact on patient safety, are also a challenge to overcome, particularly the absolute need for neuroimaging in the context of treatment decision-making (which cannot yet be replaced by some quantitative measurement of antigenic load). Therefore, guidelines need to be developed that specifically address the safety and logistical aspects of treatment in low-resource settings. The current WHO guidelines on management of NCC would represent an ideal platform for incorporating these newer aspects of NCC treatment and could be updated. The revision should include recommendations on combination therapy as first-line therapy and on the dosage and duration of corticosteroid therapy before, during and after treatment with antiparasitic medication in people with active symptomatic NCC. Making some changes based on our observations may increase efficacy and safety of NCC treatment, especially in low-resource settings, and therefore, improve health and quality of life of affected patients and their families.

## Supplementary Information

Below is the link to the electronic supplementary material.

Supplementary file1 (DOCX 649 KB)

## Data Availability

Deidentified participant data will be made available through the data repository of the Technical University of Munich upon publication of the manuscript.
