# Supplementary material for: Efficacy and safety of antiparasitic therapy for neurocysticercosis in rural Tanzania: a prospective cohort study
Source: Infection. 2023 Mar 24;51(4):1127–39. doi: 10.1007/s15010-023-02021-y (PMC10037392; doi:10.1007/s15010-023-02021-y)
Supplement: Supplementary file 1 — Supplementary file1 (DOCX 649 KB) [file 15010_2023_2021_MOESM1_ESM.docx]

S Figure 1. Treatment efficacy (cyst resolution) of albendazole monotherapy and of combination therapy in patients who participated at both the six-week and the six-month follow-up


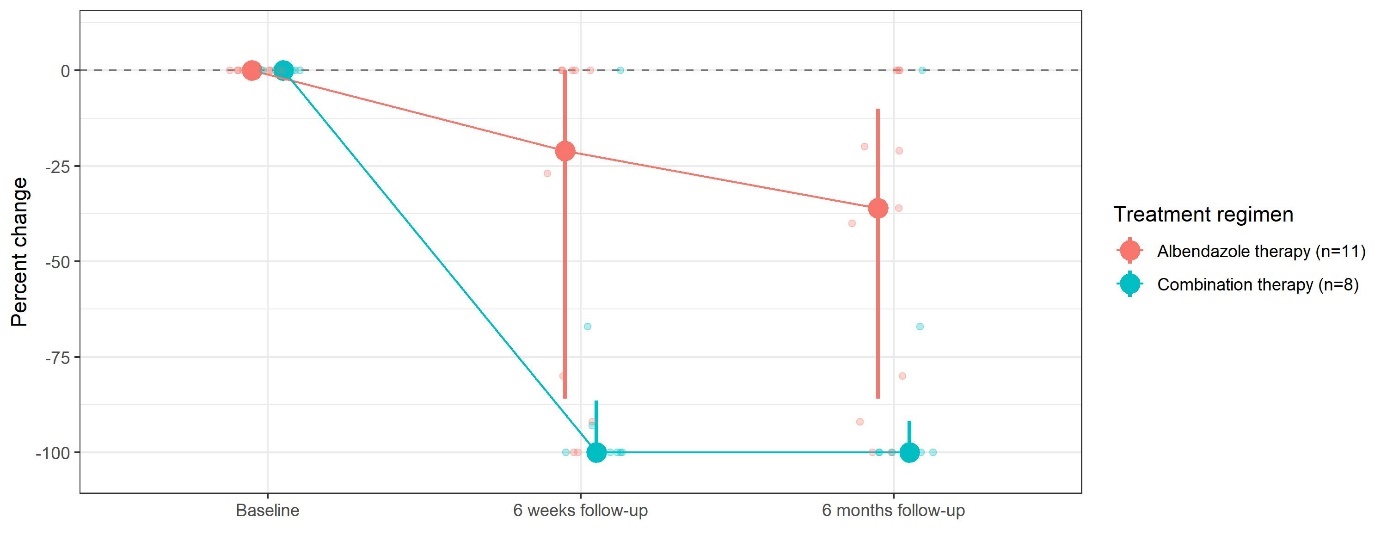


S Table 1. Cyst resolution at baseline and final follow-up in patients not treated with antiparasitic medication

|  | Baseline  N=7 | | | Long-time follow-up  N=7 | | | Lesion reduction |  |
| --- | --- | --- | --- | --- | --- | --- | --- | --- |
|  | Sum | Median [IQR] | Mean | Sum | Median [IQR] | Mean | Median (IQR) | p-value (Wilcox) |
| Total active lesions | 185 | 20 [9–47] | 26.4 | 182 | 20 [9.5–46.5] | 26.0 | 0%  (0 to 4%) | 0.37 |
| Active parenchymal lesions | 133 | 11 [7–31.5] | 19.0 | 133 | 11 [7–31.5] | 19.0 | 0%  (0 to 7%) | 1.00 |
| Active extraparenchmal lesions | 52 | 11 [2–11.5] | 7.4 | 49 | 11 [1.5–11.5] | 7.0 | 0%  (0 to 0%) | 0.41 |
| Number of scolizes | 177 | 20 [8–44.5] | 25.3 | 179 | 20 [8.5–44] | 25.6 | 0%  (0 to 4%) | 0.42 |
| Calcifications | 74 | 13 [8–13.5] | 10.6 | 67 | 13 [7.5–13] | 9.6 | 0%  (0 to 0%) | 0.42 |
| NCC with active lesions |  | 7 |  |  | 7 |  |  |  |

S Table 2. Cyst resolution between baseline and the two follow-ups in patients who were present at both the six-week and the six-month follow-up

|  | Baseline  N=11 | | | Six-week follow-up (FU)  N=11 | | | Baseline to six-week FU | Six-month FU  N=11 | | | Six-week to six-month FU |
| --- | --- | --- | --- | --- | --- | --- | --- | --- | --- | --- | --- |
|  | Sum | Median [IQR] | Mean | Sum | Median [IQR] | Mean | p-value (Wilcoxon) | Sum | Median [IQR] | Mean | p-value (Wilcoxon) |
| Total active lesions | 78 | 5  [1–8] | 7.1 | 50 | 1  [1–5] | 4.5 | 0.04 | 46 | 1  [1–3.5] | 4.2 | 0.17 |
| Active parenchymal lesions | 62 | 1  [1–6.5] | 5.6 | 40 | 1  [1–2] | 3.6 | 0.10 | 38 | 1  [1–1.5] | 3.5 | 0.36 |
| Active extraparenchymal lesions | 16 | 1  [0–2] | 1.5 | 10 | 0  [0–1] | 0.9 | 0.05 | 8 | 0  [0–0.5] | 0.7 | 0.35 |
| Number of scolices | 49 | 2  [1–4.5] | 4.5 | 39 | 1  [0–2] | 3.5 | 0.18 | 33 | 0  [0–1] | 3.0 | 0.09 |
| Calcifications | 185 | 17  [8.5–24] | 16.8 | 186 | 17  [8.5–24] | 16.9 | 1.00 | 186 | 17  [8.5–24.5] | 16.9 | 1.00 |
| NCC with active lesions |  | 11 |  |  | 10 |  |  |  | 10 |  |  |

S Table 3. Cyst resolution between baseline and final follow-up in patients who received two treatment rounds: The first with albendazole monotherapy, and the second with a combination therapy consisting of albendazole and praziquantel

|  | Baseline  N=8 | | | Long-time follow-up  Treatment round 1  N=8 | | | Lesion reduction  Treatment round 1 | | Long-time follow-up  Treatment round 2  N=8 | | | Lesion reduction  Treatment round 2 | |
| --- | --- | --- | --- | --- | --- | --- | --- | --- | --- | --- | --- | --- | --- |
|  | Sum | Median [IQR] | Mean | Sum | Median [IQR] | Mean | Median  [IQR] | p-value (Wilcoxon) | Sum | Median [IQR] | Mean | Median  [IQR] | p-value (Wilcoxon) |
| Total active lesions | 101 | 10  [7.3–13.5] | 12.6 | 67/  66^a^ | 5.5  [3.8–7.8] | 8.4/  8.2^a^ | 41%  [20 to 45%] | 0.02 | 3 | 0  [0–0.3] | 0.4 | 100%  [92 to 100%] | 0.04 |
| Active parenchymal lesions | 73 | 5  [1–12.8] | 9.1 | 56 | 4.5  [1.8–8.5] | 7.0 | 29%  [0 to 43%] | 0.06 | 1 | 0  [0–0] | 0.1 | 100%  [100 to 100%] | 0.04 |
| Active extraparenchymal lesions | 28 | 3.5  [1–4.3] | 3.5 | 11/10^a^ | 1  [0–2.3] | 1.4/1.3^a^ | 33%  [23 to 63%] | 0.03 | 2 | 0  [0–0.3] | 0.2 | 100%  [0 to 100%] | 0.18 |
| Number of scolices | 77 | 6.5  [4.3–13.0] | 9.6 | 54 | 3.5  [1.8–6.8] | 6.8 | 38%  [16 to 48%] | 0.06 | 0 | 0 | 0 | 100%  [100 to 100%] | 0.02 |
| Calcifications | 129 | 17.5  [13.5–18.5] | 16.1 | 132 | 18  [14.8–18.5] | 16.5 | 0%  [0 to 1%] | 0.79 | 138 | 18  [14.8–20.5] | 17.2 | 0%  [–10% to 0%] | 0.26 |
| NCC with active lesions |  | 8 |  |  | 8 |  |  |  |  | 2 |  |  |  |

^a^ Between six-month follow-up after albendazole monotherapy and the follow-up in July 2021, one lesion resolved. The number in (parentheses) shows the number of lesions at baseline of treatment round 2. The difference did not affect the median [IQR] reduction of lesion.

S Table 4. Quality of life assessment (QOLIE-31 and WHOQOL-BREF) before and after combination therapy (n=8)

|  | **Baseline**  **(n=8)** | **6-month follow-up**  **(n=8)** | **Difference** | **p-value** |
| --- | --- | --- | --- | --- |
|  | Median (IQR) | Median (IQR) | Median (IQR) | Paired Wilcoxon test |
| QOLIE-31(range 0 to 100) |  |  |  |  |
| Overall | 86.5 (80.1–89.4) | 94.7 (92.9–95.4) | 7.8 (5.6–10.1) | 0.02 |
| Seizure worry | 84.7 (70.3–100) | 100 (95–100) | 15.3 (0–22.2) | 0.06 |
| Overall quality of life | 75 (72.5–77.5) | 77.5 (77.5–80) | 2.5 (2.5–5) | 0.02 |
| Emotional well-being | 86 (81–92) | 92 (85–92) | 2 (0–7) | 0.53 |
| Energy/fatigue | 75 (71.3–80) | 90 (90–90) | 12.5 (10–15) | 0.01 |
| Cognitive function | 86.1 (81.1–99.2) | 100 (100–100) | 13.9 (0.8–18.3) | 0.2 |
| Medication effects | 100 (100–100) | 100 (100–100) |  |  |
| Social function | 100 (87–100) | 100 (100–100) | 0 (0–13) | 0.17 |
| WHOQOL-BREF (range 4 to 20) |  |  |  |  |
| Physical health | 15.4 (15–15.4) | 16 (16–16) | 0.6 (0.6–1) | 0.11 |
| Psychological health | 14.7 (14.7–15.3) | 16 (16–16) | 1 (0.7–1.3) | 0.01 |
| Social relationships | 13.3 (12–14.7) | 16 (16–16) | 2.7 (1.3–4) | 0.02 |
| Environment | 12 (11–12.5) | 13 (13–13.5) | 1.5 (0.6–2.4) | 0.01 |

IQR Interquartile range

S Figure 2. Systolic/diastolic blood pressure and fasting blood glucose levels over the course of treatment (albendazole monotherapy [upper row] and combination therapy [lower row])


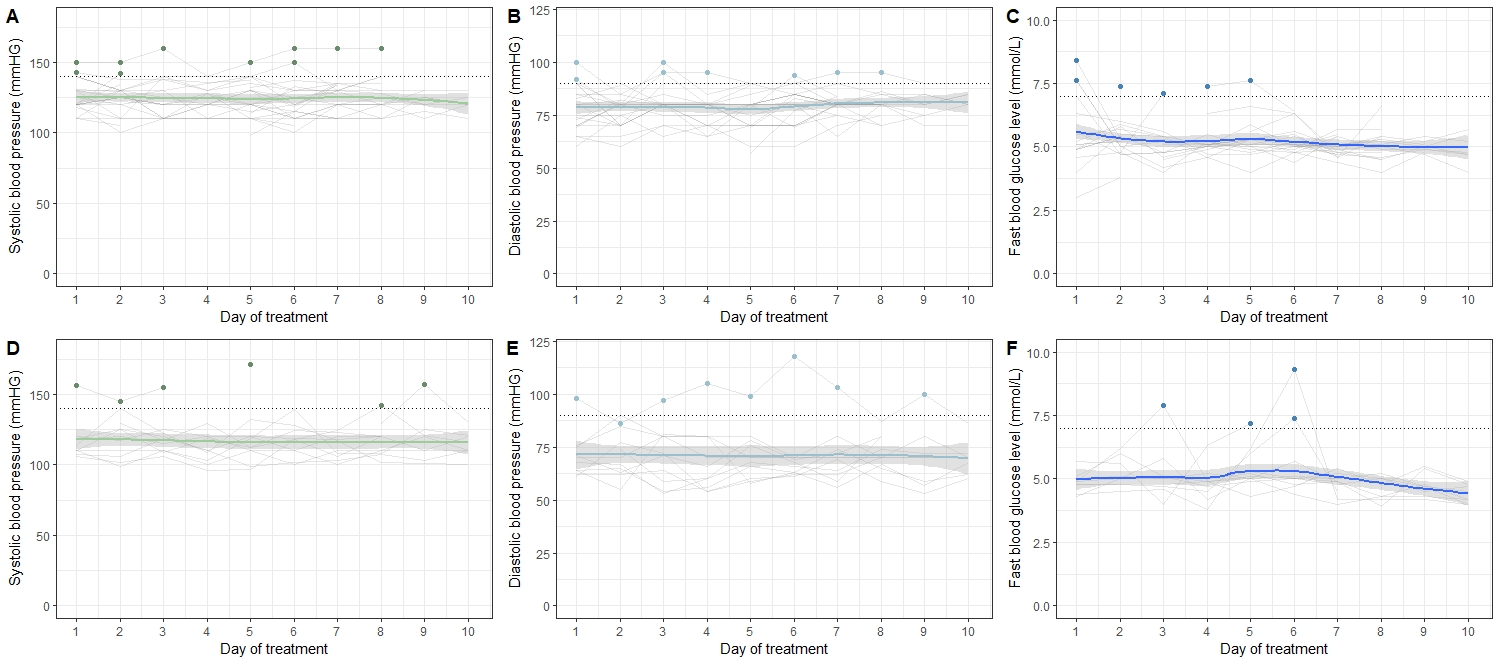


A-C Albendazole monotherapy

D-F Combination therapy

S Figure 3. Adverse events of antiparasitic treatment (panel A: albendazole monotherapy; panel B: combination therapy)


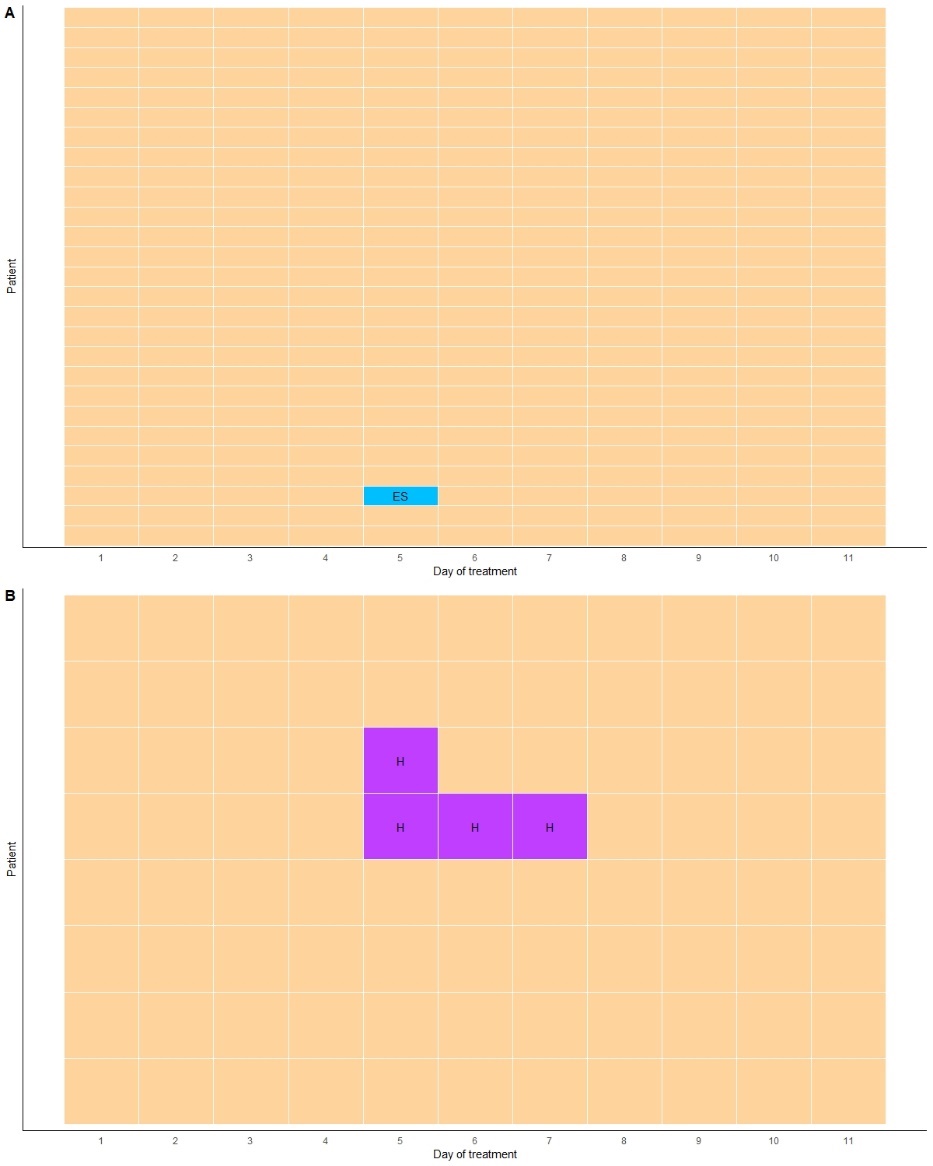


Each row represents one patient

ES Epileptic seizure

H Headache
